# Di­chloridotetra­kis­(3-meth­oxy­aniline)nickel(II)

**DOI:** 10.1107/S2414314624007764

**Published:** 2024-08-13

**Authors:** Benjamin A. Mukda, Diane A. Dickie, Mark M. Turnbull

**Affiliations:** a Carlson School of Chemistry and Biochemistry, Clark University, 950 Main St., Worcester, MA 01610, USA; b Department of Chemistry, University of Virginia, 409 McCormack Rd., Charlottesville, VA 22904, USA; Purdue University, USA

**Keywords:** nickel chloride, 3-meth­oxy­aniline, NiN_4_Cl_2_ coordination, crystal structure

## Abstract

The reaction of nickel(II) chloride with 3-meth­oxy­aniline yielded di­chlorido­tetra­kis­(3-meth­oxy­aniline)nickel(II), [NiCl_2_(C_7_H_9_NO)_4_], as yellow crystals. The Ni^II^ ion is pseudo-octa­hedral with the chloride ions *trans* to each other. The four 3-meth­oxy­aniline ligands differ primarily due to different conformations about the Ni—N bond, which also affect the hydrogen bonding. Inter­molecular N—H⋯ Cl hydrogen bonds and short Cl⋯Cl contacts between mol­ecules link them into chains parallel to the *b* axis.

## Abstract 2

The complex sits in a general position. Each Ni^II^ ion has an N_4_Cl_2_ coordination sphere. Weak hydrogen bonding exists between three of the amino groups and the chloride ions of an adjacent mol­ecule. Chains of mol­ecules, linked by the hydrogen bonding and short Cl⋯Cl contacts, are well separated by the 3-meth­oxy­aniline ligands.

## Graphical abstract

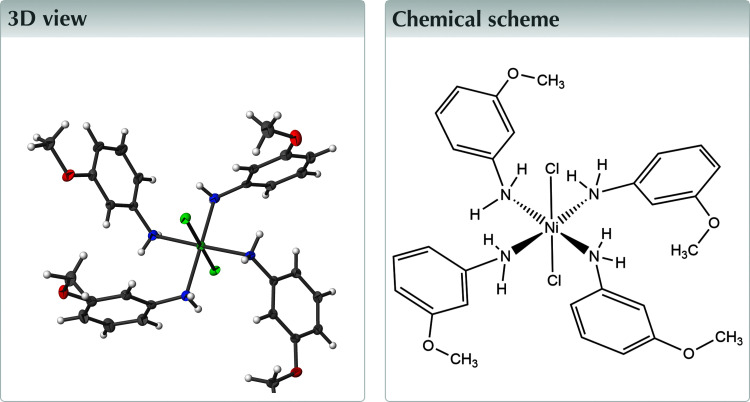

## Structure description

The structures of binary transition-metal halide complexes of aniline are varied and have been known for nearly two decades, since the report of CoCl_2_(aniline)_2_ by Burrow *et al.* (1997 ▸). Structures for compounds of the formula *MX*_2_(aniline)_2_, where *M* is a transition metal, are known for *trans*-square planar (SP) Pd (Chen *et al.*, 2002 ▸) and Cu (Low *et al.*, 2013 ▸), and tetra­hedral (*T*_d_) Zn (Khan *et al.*, 2010 ▸; Ejaz *et al.*, 2009 ▸; Rademeyer *et al.*, 2004 ▸) and Cd (Costin-Hogan *et al.*, 2008 ▸). Structures of first row transition-metal (FTM) complexes with the same general formula, FTM*X*_2_(sub-aniline)_2_ are known for substit­uents such as *o*-methyl (SP: Daniliuc *et al.*, 2023 ▸), *p*-methyl (*T*_d_: Chellali *et al.*, 2019 ▸), *p*-ethyl (*T*_d_: Govindaraj *et al.*, 2015 ▸; *T*_d_: Harmouzi *et al.*, 2017 ▸), *p*-acetyl (*T*_d_ and SP: Macek *et al.*, 2023 ▸; SP, Nemec *et al.*, 2020 ▸), *p*-bromo (*T*_d_: Subashini *et al.*, 2012*a* ▸; *T*_d_, Li: 2023 ▸), *p*-chloro (*T*_d_: Chellali *et al.*, 2019 ▸), *p*-fluoro (*T*_d_: Subashini *et al.*, 2012*b* ▸), *o*-meth­oxy, *m*-meth­oxy and *p*-meth­oxy (*T*_d_: Kupko *et al.*, 2020 ▸; *T*_d_: Amani, 2018 ▸) and *p*-carb­oxy­lic acid (*T*_d_: Rademeyer *et al.*, 2010 ▸; SP: Guedes *et al.*, 2011 ▸). Only slightly less common, but particularly favored by Ni^II^, are those structures of the formula FTM*X*_2_(sub-aniline)_2_(solvent)_2_, which include solvents such as water (Macek *et al.*, 2023 ▸; Meehan *et al.*, 2021 ▸) methanol (Meehan *et al.*, 2021 ▸), ethanol (Meehan *et al.*, 2021 ▸; Clegg & Martin, 2007 ▸) and aceto­nitrile (Fawcett *et al.*, 2005 ▸); all are *trans*-pseudo­octa­hedral (*O*_h_). A smaller number of structures have been reported with aniline and substituted aniline ligands of the formula FTM*X*_2_(sub-aniline)_4_, which include the *trans*-Oh complexes NiCl_2_(*p*-methyl­aniline)_4_ and NiBr_2_(*p*-methyl­aniline)_4_ (Meehan *et al.*, 2021 ▸) and NiI_2_(*p*-methyl­aniline)_4_ (Dhital *et al.*, 2020 ▸), again favored by six-coordinate nickel(II) complexes. In the course of our investigations of complexes of substituted aniline ligands, we have encountered one more such compound and here report the synthesis and structure of NiCl_2_(3-meth­oxy­aniline)_4_.

**Figure 1:**
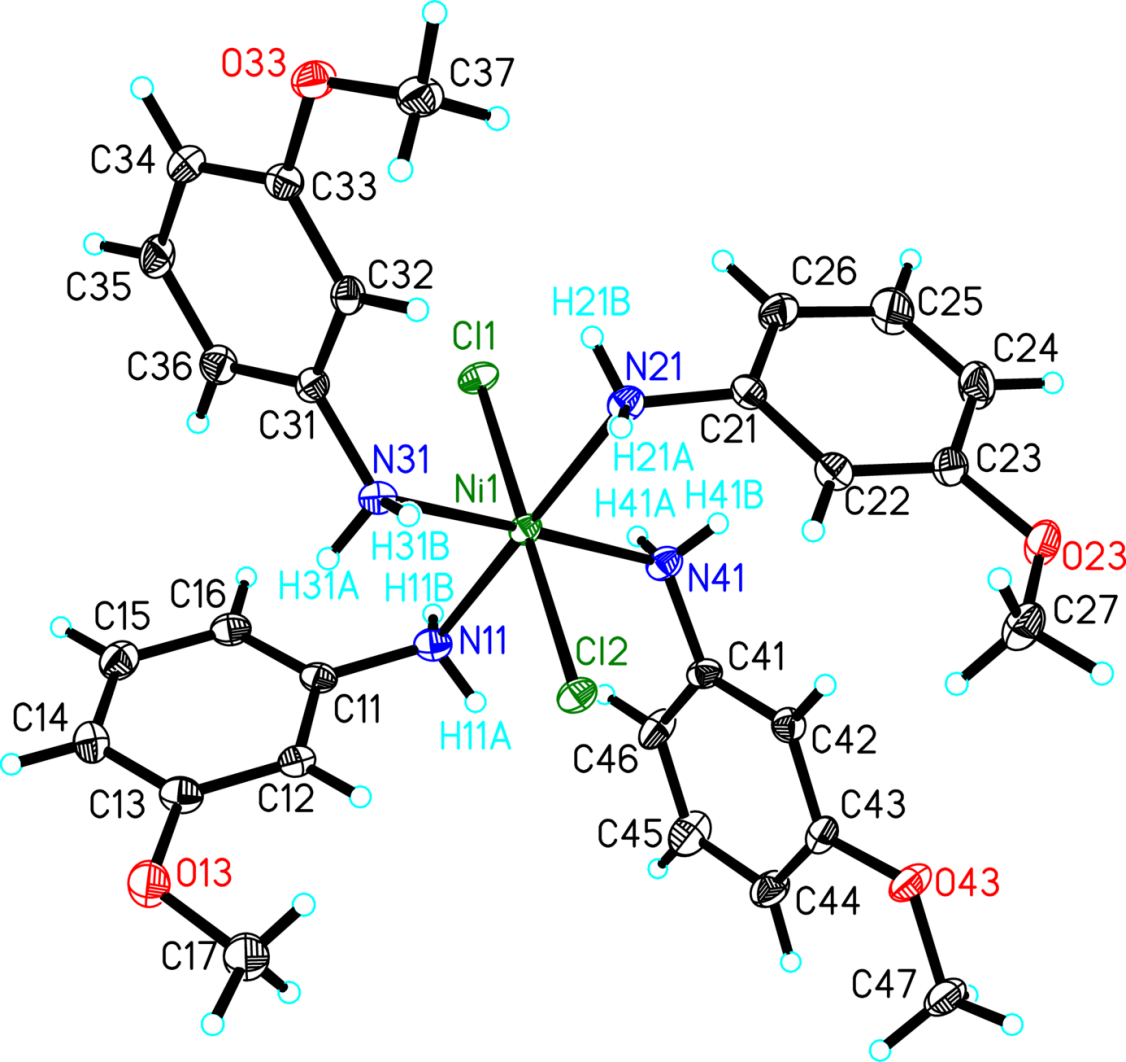
The mol­ecular structure of the title compound with displacement ellipsoids drawn at the 50% probability level. Hydrogen atoms are shown as spheres of arbitrary size. Only those hydrogen atoms whose positions were refined are labeled.

The mol­ecule is pseudo-octa­hedral with *trans*-chloride ions and all atoms lie on general crystallographic positions (Fig. 1 ▸). The Cl1—Ni1—Cl2 bond angle is nearly linear [179.8 (2)°]. The Cl—Ni—N angles range from 85.45 (5) to 93.82 (5)° while the *cis* N—Ni—N angles are similar in the range 84.3 (7) to 94.75 (7)° (Table 1 ▸). Taking the NiN_4_ atoms as the equatorial plane (mean deviation of constituent atoms = 0.0141 Å), the Ni ion lies 0 0029 Å out of the plane. One *trans*-pair of aniline ligands lie with their C—N bonds oriented nearly in that plane with angles of the C—N vector 2.6 (1)° (C11—N11) or 5.3 (1)° (C21—N21) out of the plane. Conversely, the alternate pair of aniline ligands have their C—N vectors tilted significantly out of the plane at 49.0 (1)° (C31—N31) and 44.0 (1)° (C41—N41). As expected, the aromatic rings are almost planar (mean deviation by ring: N11, 0.0115 Å; N21, 0.0212 Å; N31, 0.0028 Å; N41, 0.0222 Å). The meth­oxy groups lie very nearly in their respective ring planes as based on the torsion angles [torsion angle C*n*7—O*n*3—C*n*3—C*n*2: *n* = 1, −10.9 (3)°; 2, −7.8 (3)°; 3, −1.4 (3)°; 4, 179.32 (19)°]. The N41 ring is again unique; the conformations of the meth­oxy groups of the other three 3-meth­oxy­aniline mol­ecules all show the meth­oxy group directed toward the amino substituent, while for the N41 ring, it is rotated ∼180° and lies *anti* to the amino substituent.

It is also noteworthy that the conformations of the anisidine rings are such that three of the rings have their meth­oxy substituents tipped toward, and above, the Cl2 side of the NiN_4_ plane. The O33—C33 meth­oxy group is also tipped in that direction, but due to the orientation of the N31—C31 bond, the meth­oxy group itself lies on the opposite side of the NiN_4_ plane.

**Figure 2:**
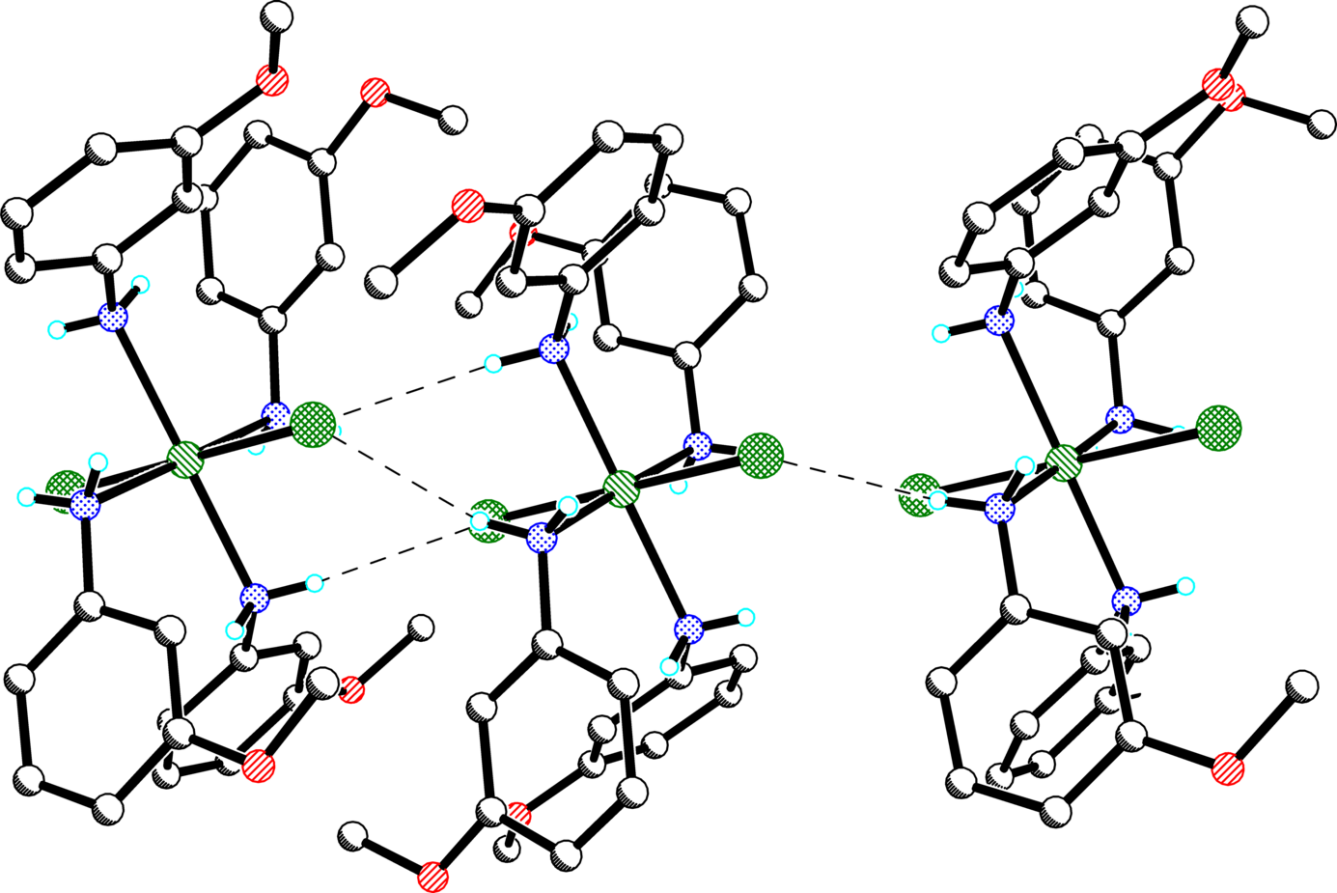
Chain formation *via* hydrogen bonding (*b* axis horizontal).

In the crystal, mol­ecules are linked into chains *via* weak N—H⋯Cl hydrogen bonds (Table 2 ▸), which results in short contacts between inversion-related chloride ions parallel to the *b* axis [*d*_Cl1⋯Cl1*A*_ = 3.725 (2) Å, angle_Ni1—Cl1⋯Cl1*A*_ = 92.4 (1)°; *d*_Cl2—Cl2*B*_ = 3.721 (2) Å, angle_Ni1—Cl2⋯Cl2*B*_ = 89.3 (1)°; symmetry codes: (*A*) = 1 − *x*, 1 − *y*, 1 − *z*; (*B*) = 1 − *x*, −*y*, 1 − *z*] (Fig. 2 ▸). The chains are well separated in both the *b*- and *c*-axis directions by the bulk of the 3-meth­oxy­aniline mol­ecules.

## Synthesis and crystallization

Synthesis: 0.5035 g of 3-meth­oxy­aniline were dissolved in 18 ml of EtOH, creating a red solution. NiCl_2_ hexa­hydrate was dissolved in 25 ml of EtOH, creating a green solution. Both solutions were heated until they began to boil, at which point the meth­oxy­aniline solution was poured into the nickel chloride solution, resulting in a peach-colored solution that quickly became cloudy. The mixture was repeatedly deca­nted to remove the majority of the precipitate over the course of two hours and then allowed to cool. The next day, a green powdery precipitate was collected using vacuum filtration and washed using DI water. The filtrate was collected and allowed to evaporate slowly. The next day, small dark-yellow crystals were observed and collected by vacuum filtration, 0.002 g (0.2%).

## Refinement

Crystal data, data collection and structure refinement details are summarized in Table 3 ▸.

## Supplementary Material

Crystal structure: contains datablock(s) I, publication_text. DOI: 10.1107/S2414314624007764/zl4076sup1.cif

CCDC reference: 2376104

Additional supporting information:  crystallographic information; 3D view; checkCIF report

## Figures and Tables

**Table 1:** Selected geometric parameters (Å, °)

Ni1—N11	2.1388 (19)	Ni1—N41	2.2056 (18)
Ni1—N21	2.1544 (19)	Ni1—Cl1	2.3658 (6)
Ni1—N31	2.1621 (18)	Ni1—Cl2	2.4051 (6)
			
N11—Ni1—N21	178.52 (8)	N11—Ni1—Cl2	89.10 (6)
N11—Ni1—N31	94.62 (7)	N21—Ni1—Cl2	92.06 (6)
N21—Ni1—N31	86.39 (7)	N31—Ni1—Cl2	85.45 (5)
N11—Ni1—N41	84.25 (7)	N41—Ni1—Cl2	93.82 (5)
N21—Ni1—N41	94.75 (7)	Cl1—Ni1—Cl2	179.86 (2)
N31—Ni1—N41	178.66 (8)	C11—N11—Ni1	120.77 (14)
N11—Ni1—Cl1	90.80 (6)	C21—N21—Ni1	116.21 (14)
N21—Ni1—Cl1	88.04 (6)	C31—N31—Ni1	125.09 (14)
N31—Ni1—Cl1	94.66 (5)	C41—N41—Ni1	123.17 (14)
N41—Ni1—Cl1	86.07 (5)		

**Table 2:** Hydrogen-bond geometry (Å, °)

*D*—H⋯*A*	*D*—H	H⋯*A*	*D*⋯*A*	*D*—H⋯*A*
N11—H11*B*⋯Cl1^i^	0.83 (2)	2.44 (3)	3.264 (2)	168 (2)
N21—H21*A*⋯Cl2^ii^	0.86 (2)	2.62 (2)	3.468 (2)	166 (2)
N31—H31*B*⋯Cl2^ii^	0.85 (2)	2.69 (2)	3.509 (2)	160 (2)

Symmetry codes: (i) 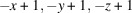; (ii) 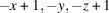.

**Table 3:** Experimental details

Crystal data	
Chemical formula	[NiCl_2_(C_7_H_9_NO)_4_]
*M*_r_	622.22
Crystal system, space group	Triclinic, *P* 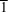
Temperature (K)	100
*a*, *b*, *c* (Å)	11.4514 (5), 12.1629 (5), 12.6920 (5)
α, β, γ (°)	67.9946 (13), 67.3255 (14), 65.8759 (14)
*V* (Å^3^)	1438.34 (11)
*Z*	2
Radiation type	Mo *K*α
μ (mm^−1^)	0.90
Crystal size (mm)	0.09 × 0.06 × 0.04

Data collection	
Diffractometer	Bruker APEXII CCD
Absorption correction	Multi-scan (*SADABS*; Krause *et al.*, 2015 ▸)
*T*_min_, *T*_max_	0.714, 0.746
No. of measured, independent and observed [*I* > 2σ(*I*)] reflections	42960, 7138, 4852
*R*_int_	0.076
(sin θ/λ)_max_ (Å^−1^)	0.667

Refinement	
*R*[*F*^2^ > 2σ(*F*^2^)], *wR*(*F*^2^), *S*	0.038, 0.089, 1.01
No. of reflections	7138
No. of parameters	380
H-atom treatment	H atoms treated by a mixture of independent and constrained refinement
Δρ_max_, Δρ_min_ (e Å^−3^)	0.39, −0.32

Computer programs: *APEX4* and*SAINT* (Bruker, 2022 ▸), *SHELXS2014* and *XP* (Sheldrick 2008 ▸) and *SHELXL2018/3* (Sheldrick, 2015 ▸).

## full crystallographic data

### Dichloridotetrakis(3-methoxyaniline)nickel(II) Crystal data

**Table:**

[NiCl_2_(C_7_H_9_NO)_4_]	*Z* = 2
*M**_r_* = 622.22	*F*(000) = 652
Triclinic, *P*1	*D*_x_ = 1.437 Mg m^−^^3^
*a* = 11.4514 (5) Å	Mo *K*α radiation, λ = 0.71073 Å
*b* = 12.1629 (5) Å	Cell parameters from 5693 reflections
*c* = 12.6920 (5) Å	θ = 2.9–27.2°
α = 67.9946 (13)°	µ = 0.90 mm^−^^1^
β = 67.3255 (14)°	*T* = 100 K
γ = 65.8759 (14)°	Plate, yellow
*V* = 1438.34 (11) Å^3^	0.09 × 0.06 × 0.04 mm

### Dichloridotetrakis(3-methoxyaniline)nickel(II) Data collection

**Table:**

Bruker APEXII CCD diffractometer	4852 reflections with *I* > 2σ(*I*)
φ and ω scans	*R*_int_ = 0.076
Absorption correction: multi-scan (SADABS; Krause *et al.*, 2015)	θ_max_ = 28.3°, θ_min_ = 2.0°
*T*_min_ = 0.714, *T*_max_ = 0.746	*h* = −15→15
42960 measured reflections	*k* = −16→16
7138 independent reflections	*l* = −16→16

### Dichloridotetrakis(3-methoxyaniline)nickel(II) Refinement

**Table:**

Refinement on *F*^2^	Primary atom site location: structure-invariant direct methods
Least-squares matrix: full	Secondary atom site location: difference Fourier map
*R*[*F*^2^ > 2σ(*F*^2^)] = 0.038	Hydrogen site location: mixed
*wR*(*F*^2^) = 0.089	H atoms treated by a mixture of independent and constrained refinement
*S* = 1.01	*w* = 1/[σ^2^(*F*_o_^2^) + (0.0334*P*)^2^ + 0.4355*P*] where *P* = (*F*_o_^2^ + 2*F*_c_^2^)/3
7138 reflections	(Δ/σ)_max_ = 0.001
380 parameters	Δρ_max_ = 0.39 e Å^−^^3^
0 restraints	Δρ_min_ = −0.32 e Å^−^^3^

### Dichloridotetrakis(3-methoxyaniline)nickel(II) Special details

**Table:**

Geometry. All esds (except the esd in the dihedral angle between two l.s. planes) are estimated using the full covariance matrix. The cell esds are taken into account individually in the estimation of esds in distances, angles and torsion angles; correlations between esds in cell parameters are only used when they are defined by crystal symmetry. An approximate (isotropic) treatment of cell esds is used for estimating esds involving l.s. planes.
Refinement. Data collection for compound 1 was carried out with a Bruker *APEX4* v2022.10–1 CCD diffractometer employing Mo—Kα radiation (λ = 0.71073 Å). The data were collected and reduced using Bruker *SMART* and *SAINT*+ software (Bruker, 2014). Absorption corrections were performed using *SADABS* (Krause, 2015). The structure was solved using *SHELXS2014* (Sheldrick, 2008) and refined using *SHELXL2018* (Sheldrick, 2015). Hydrogen atoms bonded to carbon atoms were placed geometrically and refined with fixed isotropic thermal parameters, U*_iso_*(H) = 1.2 (C). Hydrogen atoms bonded to nitrogen atoms were located in the difference map and their positions refined with fixed isotropic thermal parameters, U*_iso_*(H) = 1.2 (N) (d_N—H_ = 0.81 (2)–0.91 (2) Å). Final data collection and refinement parameters may be found in Table 2.

### Dichloridotetrakis(3-methoxyaniline)nickel(II) Fractional atomic coordinates and isotropic or equivalent isotropic displacement parameters (Å^2^)

**Table:**

	*x*	*y*	*z*	*U*_iso_*/*U*_eq_	
Ni1	0.50342 (3)	0.25347 (2)	0.48264 (2)	0.01306 (8)	
Cl1	0.64202 (5)	0.35611 (5)	0.47889 (5)	0.01674 (12)	
Cl2	0.36209 (5)	0.14962 (5)	0.48636 (5)	0.01653 (12)	
N11	0.3352 (2)	0.36558 (18)	0.58739 (17)	0.0166 (4)	
H11A	0.275 (2)	0.378 (2)	0.558 (2)	0.020*	
H11B	0.352 (2)	0.431 (2)	0.573 (2)	0.020*	
C11	0.2928 (2)	0.31967 (19)	0.71270 (19)	0.0157 (4)	
C12	0.2248 (2)	0.2323 (2)	0.75867 (19)	0.0164 (5)	
H12	0.205531	0.205855	0.707310	0.020*	
O13	0.11618 (18)	0.09999 (16)	0.93113 (14)	0.0279 (4)	
C13	0.1853 (2)	0.1843 (2)	0.8798 (2)	0.0195 (5)	
C14	0.2155 (2)	0.2202 (2)	0.9554 (2)	0.0222 (5)	
H14	0.190108	0.185492	1.038361	0.027*	
C15	0.2830 (2)	0.3071 (2)	0.9086 (2)	0.0223 (5)	
H15	0.303439	0.332402	0.960012	0.027*	
C16	0.3215 (2)	0.3580 (2)	0.7873 (2)	0.0185 (5)	
H16	0.366913	0.418434	0.755974	0.022*	
C17	0.0651 (3)	0.0796 (2)	0.8560 (2)	0.0306 (6)	
H17A	0.006179	0.158736	0.820813	0.037*	
H17B	0.014986	0.020045	0.902455	0.037*	
H17C	0.139042	0.046029	0.793178	0.037*	
N21	0.6746 (2)	0.14469 (17)	0.37458 (16)	0.0158 (4)	
H21A	0.664 (2)	0.072 (2)	0.397 (2)	0.019*	
H21B	0.741 (2)	0.143 (2)	0.391 (2)	0.019*	
C21	0.6950 (2)	0.1932 (2)	0.24955 (18)	0.0162 (5)	
O33	1.00929 (15)	−0.14771 (14)	0.62401 (13)	0.0208 (4)	
C22	0.6279 (2)	0.1668 (2)	0.19530 (19)	0.0170 (5)	
H22	0.575639	0.112201	0.239635	0.020*	
O23	0.57495 (17)	0.20291 (15)	0.01482 (13)	0.0227 (4)	
C23	0.6384 (2)	0.2214 (2)	0.07549 (19)	0.0185 (5)	
C24	0.7149 (3)	0.3015 (2)	0.0103 (2)	0.0261 (6)	
H24	0.721742	0.338922	−0.071467	0.031*	
C25	0.7802 (3)	0.3257 (2)	0.0657 (2)	0.0287 (6)	
H25	0.832512	0.380328	0.021309	0.034*	
C26	0.7717 (2)	0.2719 (2)	0.1858 (2)	0.0226 (5)	
H26	0.817848	0.289221	0.222808	0.027*	
C27	0.5077 (3)	0.1107 (2)	0.0754 (2)	0.0247 (5)	
H27A	0.569940	0.031131	0.105624	0.030*	
H27B	0.473733	0.100612	0.020716	0.030*	
H27C	0.433351	0.137241	0.141548	0.030*	
N31	0.55035 (19)	0.10066 (17)	0.63226 (16)	0.0147 (4)	
H31A	0.475 (2)	0.121 (2)	0.691 (2)	0.018*	
H31B	0.551 (2)	0.040 (2)	0.614 (2)	0.018*	
C31	0.6663 (2)	0.06435 (19)	0.67155 (18)	0.0146 (4)	
C32	0.7799 (2)	−0.02535 (19)	0.62731 (18)	0.0152 (4)	
H32	0.780233	−0.061587	0.572592	0.018*	
C33	0.8928 (2)	−0.0614 (2)	0.66390 (19)	0.0165 (5)	
C34	0.8918 (2)	−0.0100 (2)	0.74531 (19)	0.0200 (5)	
H34	0.968644	−0.035787	0.771305	0.024*	
C35	0.7780 (2)	0.0787 (2)	0.78804 (19)	0.0205 (5)	
H35	0.777267	0.114153	0.843589	0.025*	
C36	0.6646 (2)	0.1173 (2)	0.75138 (19)	0.0187 (5)	
H36	0.587098	0.179170	0.780764	0.022*	
C37	1.0107 (2)	−0.2039 (2)	0.5425 (2)	0.0223 (5)	
H37A	0.943022	−0.246875	0.579432	0.027*	
H37B	1.098606	−0.264165	0.520807	0.027*	
H37C	0.991446	−0.139306	0.471444	0.027*	
N41	0.4506 (2)	0.41029 (18)	0.33230 (17)	0.0174 (4)	
H41A	0.464 (2)	0.465 (2)	0.343 (2)	0.021*	
H41B	0.509 (2)	0.388 (2)	0.273 (2)	0.021*	
C41	0.3218 (2)	0.4570 (2)	0.31188 (19)	0.0158 (5)	
C42	0.2933 (2)	0.4009 (2)	0.25224 (18)	0.0166 (5)	
H42	0.360547	0.334924	0.219965	0.020*	
O43	0.14760 (16)	0.38059 (15)	0.17817 (14)	0.0234 (4)	
C43	0.1662 (2)	0.4410 (2)	0.23955 (19)	0.0175 (5)	
C44	0.0670 (2)	0.5362 (2)	0.2874 (2)	0.0232 (5)	
H44	−0.020672	0.562393	0.280350	0.028*	
C45	0.0980 (3)	0.5922 (2)	0.3455 (2)	0.0285 (6)	
H45	0.030717	0.658162	0.377741	0.034*	
C46	0.2242 (2)	0.5547 (2)	0.3578 (2)	0.0221 (5)	
H46	0.244053	0.595015	0.397112	0.027*	
C47	0.0172 (2)	0.4192 (2)	0.1650 (2)	0.0251 (5)	
H47A	−0.046398	0.405454	0.243101	0.030*	
H47B	0.016330	0.370547	0.118882	0.030*	
H47C	−0.007743	0.507929	0.123847	0.030*	

### Dichloridotetrakis(3-methoxyaniline)nickel(II) Atomic displacement parameters (Å^2^)

**Table:**

	*U*^11^	*U*^22^	*U*^33^	*U*^12^	*U*^13^	*U*^23^
Ni1	0.01355 (15)	0.01235 (14)	0.01499 (15)	−0.00477 (11)	−0.00524 (11)	−0.00311 (10)
Cl1	0.0156 (3)	0.0136 (3)	0.0248 (3)	−0.0050 (2)	−0.0089 (2)	−0.0049 (2)
Cl2	0.0178 (3)	0.0147 (3)	0.0210 (3)	−0.0067 (2)	−0.0081 (2)	−0.0040 (2)
N11	0.0163 (10)	0.0139 (9)	0.0203 (10)	−0.0064 (8)	−0.0050 (8)	−0.0033 (7)
C11	0.0107 (11)	0.0146 (11)	0.0188 (11)	−0.0008 (9)	−0.0029 (9)	−0.0057 (8)
C12	0.0137 (11)	0.0171 (11)	0.0196 (11)	−0.0042 (9)	−0.0056 (9)	−0.0056 (8)
O13	0.0374 (11)	0.0331 (10)	0.0220 (9)	−0.0243 (9)	−0.0084 (8)	−0.0012 (7)
C13	0.0187 (12)	0.0155 (11)	0.0240 (12)	−0.0070 (9)	−0.0049 (10)	−0.0040 (9)
C14	0.0241 (13)	0.0240 (12)	0.0173 (12)	−0.0087 (10)	−0.0059 (10)	−0.0024 (9)
C15	0.0220 (13)	0.0238 (12)	0.0242 (13)	−0.0052 (10)	−0.0089 (10)	−0.0089 (10)
C16	0.0166 (12)	0.0169 (11)	0.0239 (12)	−0.0070 (9)	−0.0058 (10)	−0.0049 (9)
C17	0.0397 (17)	0.0384 (15)	0.0250 (13)	−0.0279 (13)	−0.0076 (12)	−0.0037 (11)
N21	0.0173 (10)	0.0148 (10)	0.0178 (10)	−0.0072 (8)	−0.0061 (8)	−0.0029 (7)
C21	0.0142 (11)	0.0167 (11)	0.0163 (11)	−0.0029 (9)	−0.0023 (9)	−0.0067 (8)
O33	0.0160 (9)	0.0248 (9)	0.0243 (9)	−0.0022 (7)	−0.0067 (7)	−0.0124 (7)
C22	0.0178 (12)	0.0150 (11)	0.0179 (11)	−0.0064 (9)	−0.0029 (9)	−0.0047 (8)
O23	0.0293 (10)	0.0268 (9)	0.0178 (8)	−0.0128 (8)	−0.0097 (7)	−0.0036 (7)
C23	0.0187 (13)	0.0201 (12)	0.0184 (12)	−0.0051 (10)	−0.0046 (10)	−0.0083 (9)
C24	0.0325 (15)	0.0320 (14)	0.0154 (12)	−0.0174 (12)	−0.0047 (10)	−0.0015 (10)
C25	0.0338 (16)	0.0341 (15)	0.0227 (13)	−0.0236 (12)	−0.0036 (11)	−0.0017 (11)
C26	0.0224 (13)	0.0291 (13)	0.0226 (12)	−0.0140 (11)	−0.0073 (10)	−0.0054 (10)
C27	0.0318 (15)	0.0255 (13)	0.0250 (13)	−0.0136 (11)	−0.0137 (11)	−0.0038 (10)
N31	0.0132 (10)	0.0129 (9)	0.0184 (10)	−0.0037 (8)	−0.0042 (8)	−0.0048 (7)
C31	0.0160 (11)	0.0138 (11)	0.0138 (10)	−0.0069 (9)	−0.0052 (9)	0.0002 (8)
C32	0.0178 (12)	0.0150 (11)	0.0143 (11)	−0.0057 (9)	−0.0047 (9)	−0.0043 (8)
C33	0.0152 (12)	0.0156 (11)	0.0170 (11)	−0.0047 (9)	−0.0037 (9)	−0.0034 (8)
C34	0.0181 (12)	0.0254 (12)	0.0192 (12)	−0.0070 (10)	−0.0080 (9)	−0.0053 (9)
C35	0.0253 (13)	0.0244 (12)	0.0179 (12)	−0.0086 (10)	−0.0073 (10)	−0.0094 (9)
C36	0.0187 (12)	0.0180 (11)	0.0181 (11)	−0.0027 (9)	−0.0047 (9)	−0.0069 (9)
C37	0.0167 (12)	0.0255 (13)	0.0268 (13)	−0.0012 (10)	−0.0053 (10)	−0.0155 (10)
N41	0.0183 (11)	0.0167 (10)	0.0197 (10)	−0.0073 (8)	−0.0075 (8)	−0.0029 (8)
C41	0.0142 (11)	0.0150 (11)	0.0172 (11)	−0.0058 (9)	−0.0063 (9)	0.0003 (8)
C42	0.0165 (12)	0.0162 (11)	0.0163 (11)	−0.0040 (9)	−0.0045 (9)	−0.0045 (8)
O43	0.0202 (9)	0.0271 (9)	0.0312 (9)	−0.0043 (7)	−0.0129 (7)	−0.0136 (7)
C43	0.0224 (13)	0.0173 (11)	0.0165 (11)	−0.0080 (10)	−0.0092 (9)	−0.0023 (8)
C44	0.0177 (13)	0.0260 (13)	0.0269 (13)	−0.0004 (10)	−0.0112 (10)	−0.0098 (10)
C45	0.0256 (14)	0.0267 (13)	0.0359 (15)	0.0053 (11)	−0.0149 (12)	−0.0190 (11)
C46	0.0247 (13)	0.0210 (12)	0.0268 (13)	−0.0021 (10)	−0.0153 (11)	−0.0095 (10)
C47	0.0238 (14)	0.0313 (14)	0.0282 (13)	−0.0106 (11)	−0.0132 (11)	−0.0074 (10)

### Dichloridotetrakis(3-methoxyaniline)nickel(II) Geometric parameters (Å, º)

**Table:**

Ni1—N11	2.1388 (19)	C25—H25	0.9500
Ni1—N21	2.1544 (19)	C26—H26	0.9500
Ni1—N31	2.1621 (18)	C27—H27A	0.9800
Ni1—N41	2.2056 (18)	C27—H27B	0.9800
Ni1—Cl1	2.3658 (6)	C27—H27C	0.9800
Ni1—Cl2	2.4051 (6)	N31—C31	1.440 (3)
N11—C11	1.428 (3)	N31—H31A	0.91 (2)
N11—H11A	0.85 (2)	N31—H31B	0.85 (2)
N11—H11B	0.83 (2)	C31—C36	1.381 (3)
C11—C16	1.385 (3)	C31—C32	1.391 (3)
C11—C12	1.392 (3)	C32—C33	1.388 (3)
C12—C13	1.384 (3)	C32—H32	0.9500
C12—H12	0.9500	C33—C34	1.388 (3)
O13—C13	1.367 (3)	C34—C35	1.379 (3)
O13—C17	1.428 (3)	C34—H34	0.9500
C13—C14	1.387 (3)	C35—C36	1.388 (3)
C14—C15	1.382 (3)	C35—H35	0.9500
C14—H14	0.9500	C36—H36	0.9500
C15—C16	1.390 (3)	C37—H37A	0.9800
C15—H15	0.9500	C37—H37B	0.9800
C16—H16	0.9500	C37—H37C	0.9800
C17—H17A	0.9800	N41—C41	1.436 (3)
C17—H17B	0.9800	N41—H41A	0.81 (2)
C17—H17C	0.9800	N41—H41B	0.83 (2)
N21—C21	1.430 (3)	C41—C42	1.381 (3)
N21—H21A	0.86 (2)	C41—C46	1.390 (3)
N21—H21B	0.85 (2)	C42—C43	1.387 (3)
C21—C26	1.379 (3)	C42—H42	0.9500
C21—C22	1.393 (3)	O43—C43	1.370 (3)
O33—C33	1.368 (3)	O43—C47	1.428 (3)
O33—C37	1.431 (3)	C43—C44	1.385 (3)
C22—C23	1.391 (3)	C44—C45	1.383 (3)
C22—H22	0.9500	C44—H44	0.9500
O23—C23	1.366 (3)	C45—C46	1.380 (3)
O23—C27	1.430 (3)	C45—H45	0.9500
C23—C24	1.391 (3)	C46—H46	0.9500
C24—C25	1.371 (3)	C47—H47A	0.9800
C24—H24	0.9500	C47—H47B	0.9800
C25—C26	1.397 (3)	C47—H47C	0.9800
			
N11—Ni1—N21	178.52 (8)	C21—C26—H26	120.6
N11—Ni1—N31	94.62 (7)	C25—C26—H26	120.6
N21—Ni1—N31	86.39 (7)	O23—C27—H27A	109.5
N11—Ni1—N41	84.25 (7)	O23—C27—H27B	109.5
N21—Ni1—N41	94.75 (7)	H27A—C27—H27B	109.5
N31—Ni1—N41	178.66 (8)	O23—C27—H27C	109.5
N11—Ni1—Cl1	90.80 (6)	H27A—C27—H27C	109.5
N21—Ni1—Cl1	88.04 (6)	H27B—C27—H27C	109.5
N31—Ni1—Cl1	94.66 (5)	C31—N31—Ni1	125.09 (14)
N41—Ni1—Cl1	86.07 (5)	C31—N31—H31A	110.5 (15)
N11—Ni1—Cl2	89.10 (6)	Ni1—N31—H31A	100.9 (14)
N21—Ni1—Cl2	92.06 (6)	C31—N31—H31B	109.2 (16)
N31—Ni1—Cl2	85.45 (5)	Ni1—N31—H31B	101.4 (16)
N41—Ni1—Cl2	93.82 (5)	H31A—N31—H31B	109 (2)
Cl1—Ni1—Cl2	179.86 (2)	C36—C31—C32	120.8 (2)
C11—N11—Ni1	120.77 (14)	C36—C31—N31	120.7 (2)
C11—N11—H11A	109.6 (16)	C32—C31—N31	118.46 (19)
Ni1—N11—H11A	100.8 (16)	C33—C32—C31	119.3 (2)
C11—N11—H11B	107.7 (16)	C33—C32—H32	120.3
Ni1—N11—H11B	106.1 (17)	C31—C32—H32	120.3
H11A—N11—H11B	112 (2)	O33—C33—C32	123.3 (2)
C16—C11—C12	120.4 (2)	O33—C33—C34	116.24 (19)
C16—C11—N11	121.1 (2)	C32—C33—C34	120.4 (2)
C12—C11—N11	118.4 (2)	C35—C34—C33	119.3 (2)
C13—C12—C11	119.5 (2)	C35—C34—H34	120.4
C13—C12—H12	120.3	C33—C34—H34	120.4
C11—C12—H12	120.3	C34—C35—C36	121.2 (2)
C13—O13—C17	116.54 (18)	C34—C35—H35	119.4
O13—C13—C12	122.6 (2)	C36—C35—H35	119.4
O13—C13—C14	116.7 (2)	C31—C36—C35	119.0 (2)
C12—C13—C14	120.7 (2)	C31—C36—H36	120.5
C15—C14—C13	119.2 (2)	C35—C36—H36	120.5
C15—C14—H14	120.4	O33—C37—H37A	109.5
C13—C14—H14	120.4	O33—C37—H37B	109.5
C14—C15—C16	121.0 (2)	H37A—C37—H37B	109.5
C14—C15—H15	119.5	O33—C37—H37C	109.5
C16—C15—H15	119.5	H37A—C37—H37C	109.5
C11—C16—C15	119.2 (2)	H37B—C37—H37C	109.5
C11—C16—H16	120.4	C41—N41—Ni1	123.17 (14)
C15—C16—H16	120.4	C41—N41—H41A	109.1 (18)
O13—C17—H17A	109.5	Ni1—N41—H41A	101.2 (17)
O13—C17—H17B	109.5	C41—N41—H41B	109.4 (17)
H17A—C17—H17B	109.5	Ni1—N41—H41B	104.0 (17)
O13—C17—H17C	109.5	H41A—N41—H41B	109 (2)
H17A—C17—H17C	109.5	C42—C41—C46	120.3 (2)
H17B—C17—H17C	109.5	C42—C41—N41	120.1 (2)
C21—N21—Ni1	116.21 (14)	C46—C41—N41	119.6 (2)
C21—N21—H21A	109.8 (15)	C41—C42—C43	119.9 (2)
Ni1—N21—H21A	105.2 (16)	C41—C42—H42	120.1
C21—N21—H21B	107.8 (16)	C43—C42—H42	120.1
Ni1—N21—H21B	104.7 (16)	C43—O43—C47	116.84 (18)
H21A—N21—H21B	113 (2)	O43—C43—C44	123.7 (2)
C26—C21—C22	120.7 (2)	O43—C43—C42	115.7 (2)
C26—C21—N21	120.4 (2)	C44—C43—C42	120.6 (2)
C22—C21—N21	118.6 (2)	C45—C44—C43	118.7 (2)
C33—O33—C37	116.88 (17)	C45—C44—H44	120.7
C23—C22—C21	119.3 (2)	C43—C44—H44	120.7
C23—C22—H22	120.4	C46—C45—C44	121.7 (2)
C21—C22—H22	120.4	C46—C45—H45	119.2
C23—O23—C27	117.27 (17)	C44—C45—H45	119.2
O23—C23—C24	115.8 (2)	C45—C46—C41	119.0 (2)
O23—C23—C22	123.7 (2)	C45—C46—H46	120.5
C24—C23—C22	120.5 (2)	C41—C46—H46	120.5
C25—C24—C23	119.1 (2)	O43—C47—H47A	109.5
C25—C24—H24	120.5	O43—C47—H47B	109.5
C23—C24—H24	120.5	H47A—C47—H47B	109.5
C24—C25—C26	121.6 (2)	O43—C47—H47C	109.5
C24—C25—H25	119.2	H47A—C47—H47C	109.5
C26—C25—H25	119.2	H47B—C47—H47C	109.5
C21—C26—C25	118.8 (2)		
			
Ni1—N11—C11—C16	102.6 (2)	Ni1—N31—C31—C36	88.5 (2)
Ni1—N11—C11—C12	−75.6 (2)	Ni1—N31—C31—C32	−91.6 (2)
C16—C11—C12—C13	0.3 (3)	C36—C31—C32—C33	−0.2 (3)
N11—C11—C12—C13	178.5 (2)	N31—C31—C32—C33	179.85 (19)
C17—O13—C13—C12	−10.9 (3)	C37—O33—C33—C32	−1.4 (3)
C17—O13—C13—C14	169.4 (2)	C37—O33—C33—C34	178.35 (19)
C11—C12—C13—O13	178.8 (2)	C31—C32—C33—O33	−179.19 (19)
C11—C12—C13—C14	−1.5 (3)	C31—C32—C33—C34	1.1 (3)
O13—C13—C14—C15	−178.7 (2)	O33—C33—C34—C35	179.2 (2)
C12—C13—C14—C15	1.5 (4)	C32—C33—C34—C35	−1.0 (3)
C13—C14—C15—C16	−0.4 (4)	C33—C34—C35—C36	0.2 (3)
C12—C11—C16—C15	0.8 (3)	C32—C31—C36—C35	−0.6 (3)
N11—C11—C16—C15	−177.4 (2)	N31—C31—C36—C35	179.3 (2)
C14—C15—C16—C11	−0.8 (4)	C34—C35—C36—C31	0.7 (3)
Ni1—N21—C21—C26	90.7 (2)	Ni1—N41—C41—C42	−84.4 (2)
Ni1—N21—C21—C22	−84.6 (2)	Ni1—N41—C41—C46	92.3 (2)
C26—C21—C22—C23	−0.3 (3)	C46—C41—C42—C43	−0.9 (3)
N21—C21—C22—C23	174.9 (2)	N41—C41—C42—C43	175.75 (19)
C27—O23—C23—C24	173.2 (2)	C47—O43—C43—C44	−0.5 (3)
C27—O23—C23—C22	−7.8 (3)	C47—O43—C43—C42	179.32 (19)
C21—C22—C23—O23	−179.01 (19)	C41—C42—C43—O43	179.42 (19)
C21—C22—C23—C24	0.0 (3)	C41—C42—C43—C44	−0.7 (3)
O23—C23—C24—C25	179.2 (2)	O43—C43—C44—C45	−178.6 (2)
C22—C23—C24—C25	0.2 (4)	C42—C43—C44—C45	1.5 (3)
C23—C24—C25—C26	0.0 (4)	C43—C44—C45—C46	−0.7 (4)
C22—C21—C26—C25	0.5 (3)	C44—C45—C46—C41	−0.9 (4)
N21—C21—C26—C25	−174.7 (2)	C42—C41—C46—C45	1.7 (3)
C24—C25—C26—C21	−0.3 (4)	N41—C41—C46—C45	−175.0 (2)

### Dichloridotetrakis(3-methoxyaniline)nickel(II) Hydrogen-bond geometry (Å, º)

**Table:**

*D*—H···*A*	*D*—H	H···*A*	*D*···*A*	*D*—H···*A*
N11—H11*B*···Cl1^i^	0.83 (2)	2.44 (3)	3.264 (2)	168 (2)
N21—H21*A*···Cl2^ii^	0.86 (2)	2.62 (2)	3.468 (2)	166 (2)
N31—H31*B*···Cl2^ii^	0.85 (2)	2.69 (2)	3.509 (2)	160 (2)

Symmetry codes: (i) −*x*+1, −*y*+1, −*z*+1; (ii) −*x*+1, −*y*, −*z*+1.
